# Supplementary figures and images for: Vestigial mediates the effect of insulin signaling pathway on wing-morph switching in planthoppers
Source: PLoS Genet. 2021 Feb 9;17(2):e1009312. doi: 10.1371/journal.pgen.1009312 (PMC7899339; doi:10.1371/journal.pgen.1009312)

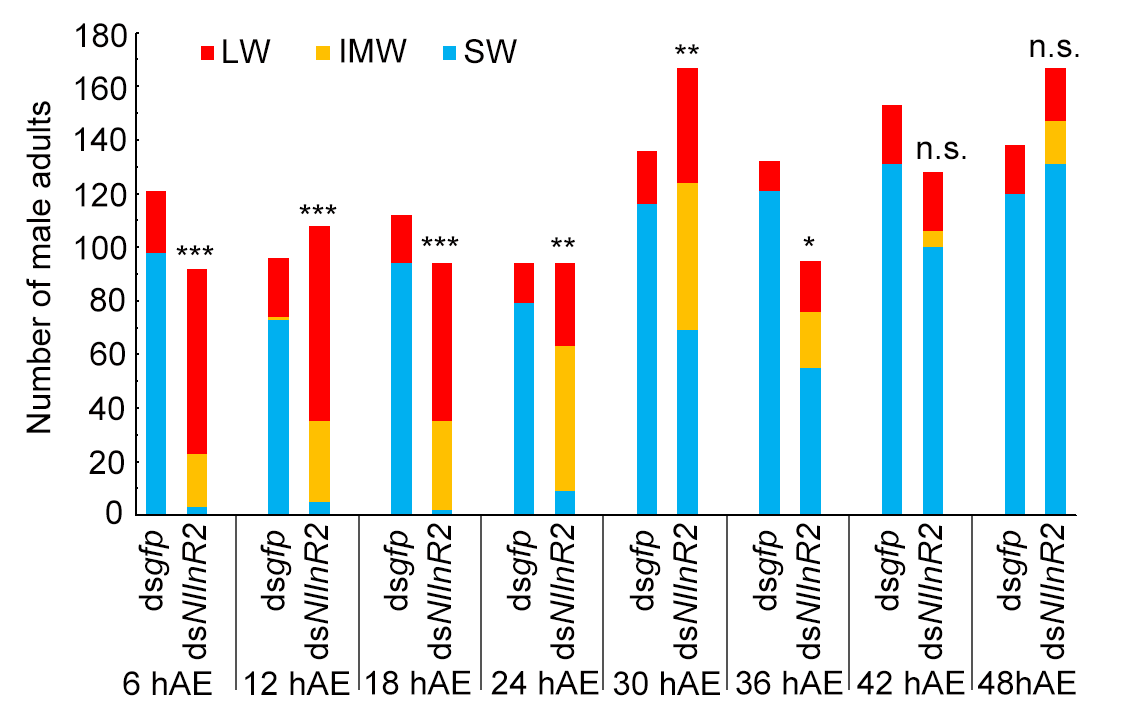

Supplement: S1 Fig — Fifth-instar male nymphs collected at designated time (6, 12, 18, 24, 30, 36, 42, and 48 hAE) were microinjected with dsNlInR2 or dsgfp. hAE, hours after ecdysis. SW, short-winged. IMW, intermediate-size wings. LW, long-winged. Non-significant (n.s.) and significant (*P < 0.05, ***P < 0.01, ***P < 0.001, Pearson’s χ2 test) differences from the control group (dsgfp) are indicated. (TIF) [file pgen.1009312.s001.tif]

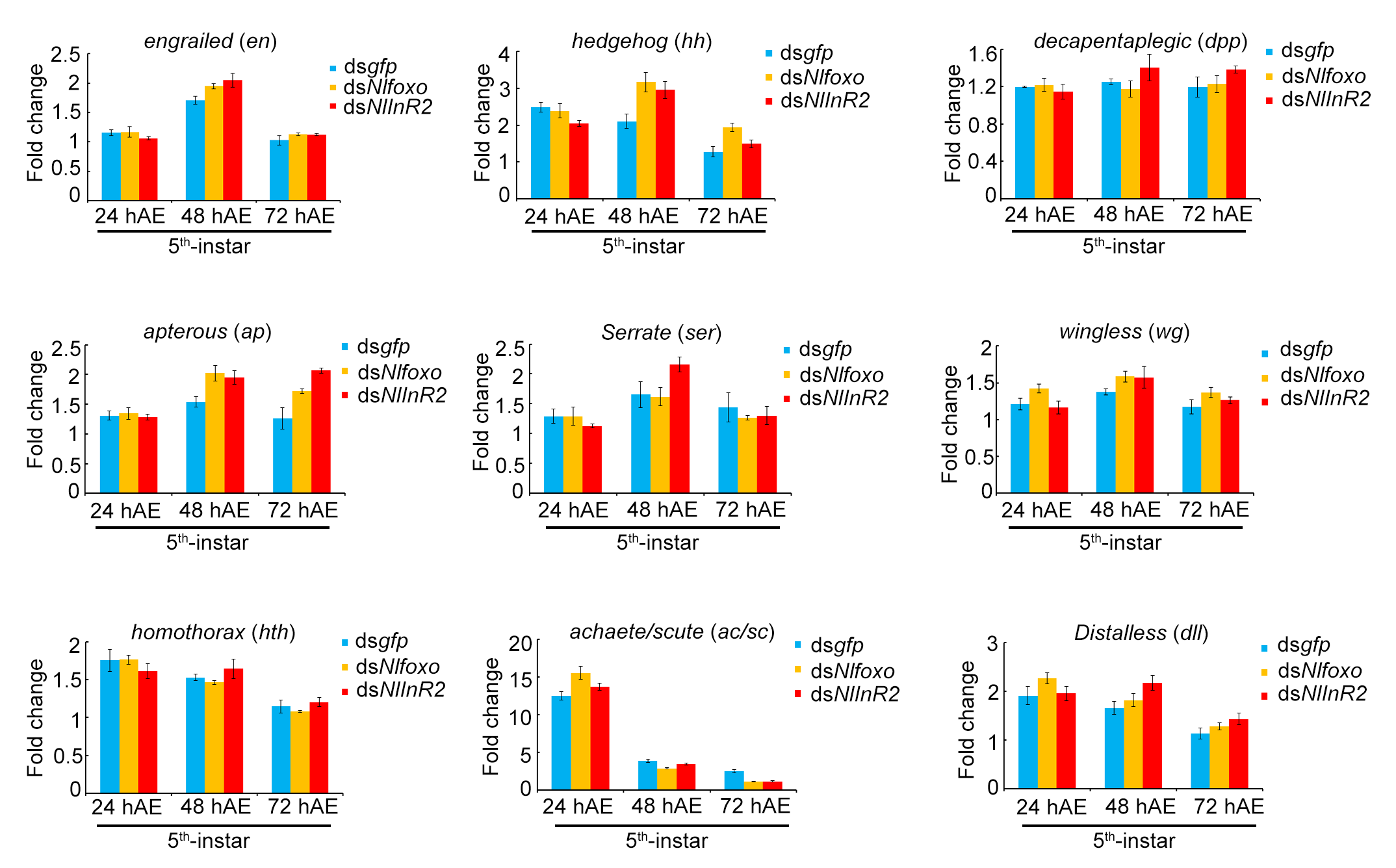

Supplement: S2 Fig — Third-instar SW-destined nymphs were microinjected with approximately 75 ng of dsNlInR2 or dsNlfoxo. Total RNA was isolated when nymphs (n = 15 for each biological replicate) reached the 24, 48, and 72 hAE fifth-instar. First-strand cDNA was synthesized, and the expression of wing-patterning genes was compared to that in the control groups (dsgfp treatment) via qRT-PCR. Bars represent mean ± s.e.m. derived from three independent biological replicates. Statistical comparisons between two groups were performed using a two-tailed Student’s t-test (*P < 0.05, **P < 0.01, and **P < 0.01). (TIF) [file pgen.1009312.s002.tif]

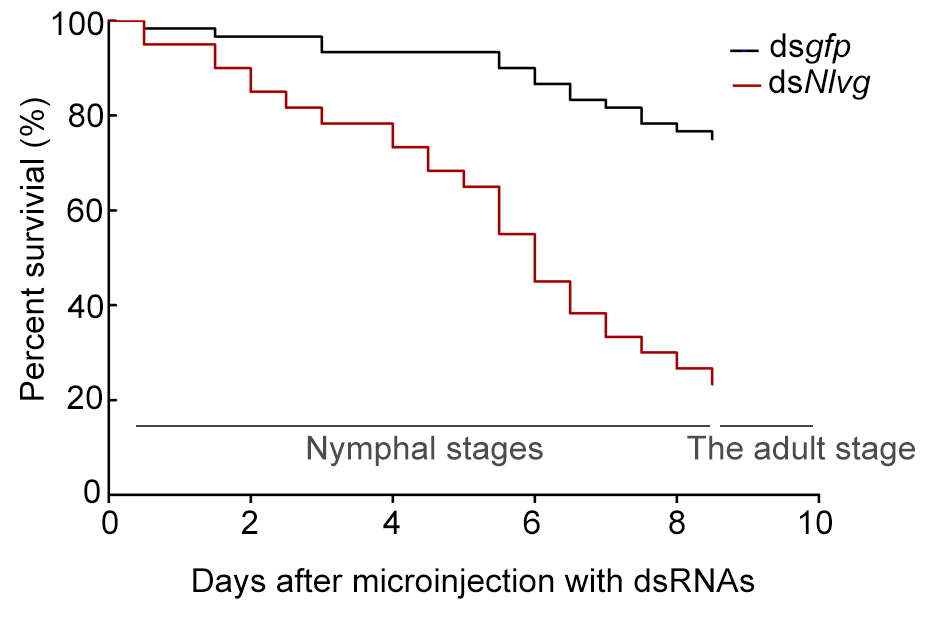

Supplement: S3 Fig — Third-instar nymphs were injected with dsNlvg (n = 60) or dsgfp (n = 60), and surviving BPHs were monitored every 12 h. dsNlvg treatment led to higher mortality relative to the dsgfp treatment (log-rank Mantel-Cox test, P < 0.001). (TIF) [file pgen.1009312.s003.tif]

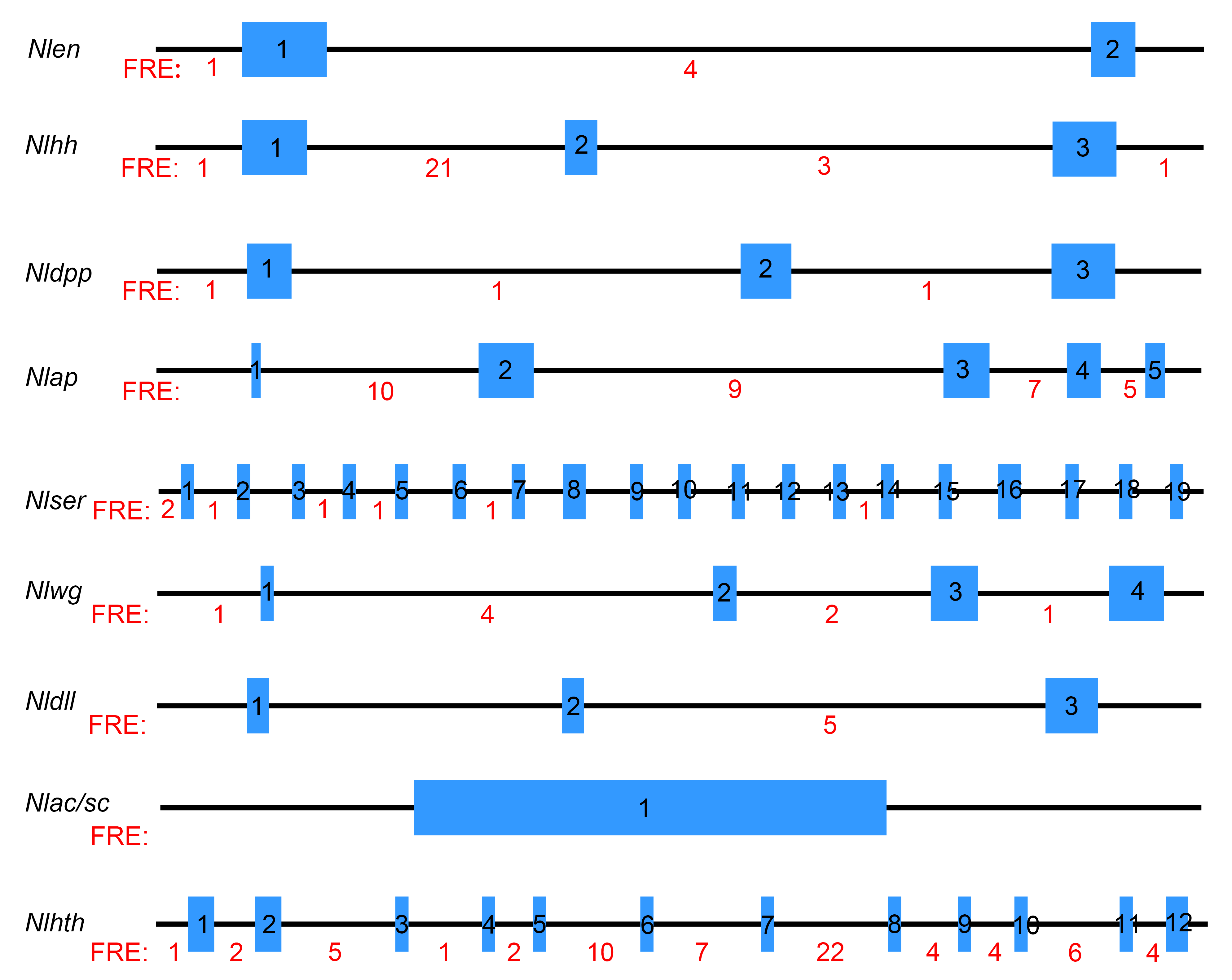

Supplement: S4 Fig — Exons were indicated by boxes in blue. The number of FoxO recognition element (FRE) containing a FoxO consensus binding site (TGTTTAC) was labeled in red number. (TIF) [file pgen.1009312.s004.tif]

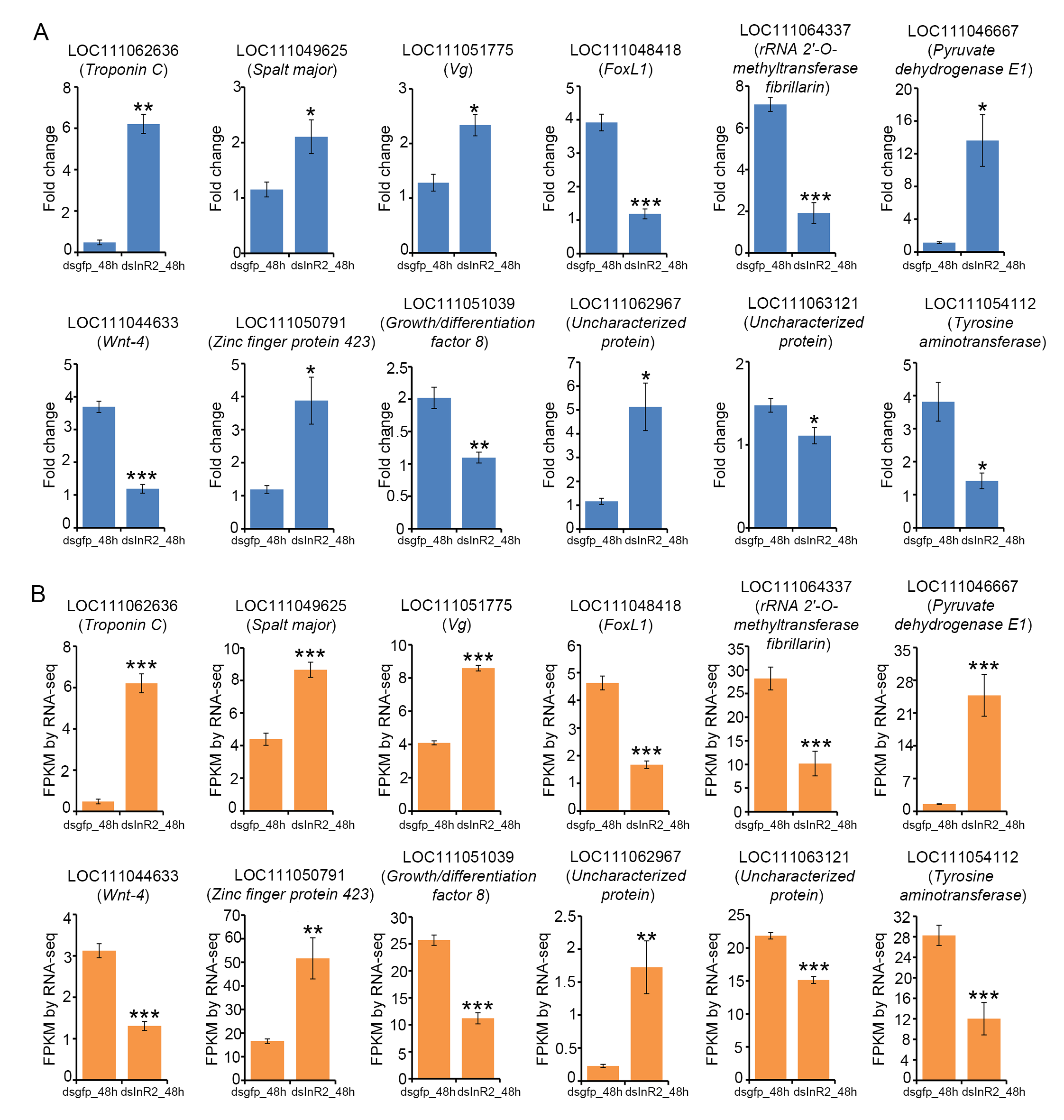

Supplement: S5 Fig — (A) Total RNA was isolated from fifth instar nymphs at 48 hAE previously treated with dsNlInR2 or dsgfp. First-strand cDNA was synthesized, and the expression of each gene was compared to that in the control groups (dsgfp treatment) via qRT-PCR. (B) The number of fragments per kilobase of transcript sequence per millions base pairs sequenced (FPKM) showed by RNA-seq. Bars represent mean ± s.e.m. derived from three independent biological replicates. Statistical comparisons between two groups were performed using a two-tailed Student’s t-test (*P < 0.05, **P < 0.01, and ***P < 0.001). (TIF) [file pgen.1009312.s005.tif]
