# Supplementary material for: Vestigial mediates the effect of insulin signaling pathway on wing-morph switching in planthoppers
Source: PLoS Genet. 2021 Feb 9;17(2):e1009312. doi: 10.1371/journal.pgen.1009312 (PMC7899339; doi:10.1371/journal.pgen.1009312)
Supplement: S1 Data — (DOCX) [file pgen.1009312.s012.docx]

**S1 Data. Transcriptomic analysis of wing buds.**

**Comparison of transcriptional profiles between dsInR2_48h and dsgfp_48h** Because only one DEG was identified in dsInR2_24h versus dsgfp_24h, we focused on the comparative transcriptomic analysis in dsInR2_48h and dsgfp_48h. We constructed six cDNA libraries from dsInR2_48h and dsgfp_48h with three biological replicates each. For each cDNA library, at least 51 million raw reads with 0.02 error rate were generated via Illumina Hiseq platform. After removing low-quality reads, adaptors, and unknown base (N) reads, total 313,889,042 clean reads were produced from 6 cDNA libraries (S2 Table). The mapping rate of clean reads against *N. lugens* reference genome ranged from 73.23% to 76.73% (S2 Table). Clean reads were mapped to 23,916 genes, including 21,455 annotated genes and 2,461 novel genes. To assess the reproducibility among three biological replicates, we calculated the pairwise correlations between every two samples in the same group. The results showed a very high Pearson correlation coefficients (R^2^ > 0.96) among three biological replicates, indicating the RNA-seq data were reliable.

Using adjusted *P*-value < 0.05 as a parameter, we identified 223 genes in down-regulated DEGs and 76 genes in up-regulated DEGs (S3 Table). The 299 DEGs account for a small percentage of all mapped genes (1.25%, 299 out of 23,916 genes).

**Functional enrichment analysis of DEGs**

To better understand the regulatory mechanisms by *Nl*InR2, we performed GO terms and KEGG pathway enrichment analysis of DEGs in dsInR2_48h versus dsgfp_48h. In GO classification analysis, 74.6% (223 out of 299) DEGs were assigned to 1,174 GO subterms which included 687 subterms in biological process category, 151 subterms in cellular component category, and 336 subterms in molecular function category (S4 Table). However, only 5 GO subterms were significantly enriched with the adjusted *P*-value < 0.05, including “cellular biogenic amine metabolic process” (GO: 0006576, 10 DEGs), “cellular amine metabolic process” (GO: 0044106, 10 DEGs), “amine metabolic process” (GO: 0009308, 10 DEGs), “cofactor binding” (GO: 0048037, 20 DEGs), and “GTPase activity” (GO: 0003924, 17 DEGs). Notably, all these significantly enriched GO subterms were involved in metabolism, and most DEGs mapped these subterms were down-regulated. In addition, we noticed that GO subterms of “non-coding RNA (ncRNA) processing” (GO: 0034470) and “ncRNA metabolic process” (GO: 0034660) were enriched under the *P*-value < 0.005.

In the KEGG pathway enrichment analysis, 72 out of 299 DEGs could categorized into 60 pathways (S5 Table). The top 10 enriched pathway were metabolic pathways (dme01100, 42 DEGs), Valine, leucine and isoleucine degradation (dme00280, 10 DEGs), ECM-receptor interaction (dme04512, 8 genes), Biosynthesis of amino acids (dme01230, 8 DEGs), Carbon metabolism (dme01200, 8 DEGs), Fatty acid metabolism (dme01212, 5 DEGs), Starch and sucrose metabolism (dme00500, 5 DEGs), Fatty acid elongation (dme00062, 4 DEGs), Cysteine and methionine metabolism (dme00270, 4 DEGs), and Glycine, serine and threonine metabolism (dme00260, 4 DEGs). Among the top 10 enriched pathways for DEGs, 90% were related to metabolism. We also noticed that 4 DEGs were assigned to three signaling pathways, including Hedgehog signaling pathway (dme04340, 2 DEGs), mTOR signaling pathway (dme04150, 1 DEGs), Hippo signaling pathway (dme04391, 1 DEGs). In addition, 2 of the 223 down-regulated DEGs were annotated as *cytochrome P450 302a1* (*CYP302A1*, log2foldchage = -1.26) and *Juvenile hormone epoxide hydrolase* (*JHEH*, log2foldchang = -0.68), which were assigned to Insect hormone biosynthesis (dme00981).

**Expression of wing-patterning genes**

Given that knockdown of *NlInR2* can redirect wing commitment from SW to LW, we especially investigated the expression levels of putative wing-patterning genes. Based on the functional annotation and tBlastn results, 15 putative wing-patterning genes homologous to those of *D. melanogaster*, *Tribolium castaneum*, and *Acyrthosiphon pisum* were identified in the RNA-seq database (S6 Table). Comparative transcriptomic analysis showed that all the genes examined except for *Nlvg* had a comparable expression between dsInR2_48h and dsgfp_48h. These results indicate that *Nlvg* is selectively regulated by *Nl*InR2 in the fifth-instar stage.

**Validation of RNA-seq data**

To validate RNA-seq results, we picked 12 DEGs for qRT-PCR analysis, which includes *Nlvg*, LOC111062636, LOC111049625, LOC111048418, LOC111044633, LOC111064377, LOC111046667, LOC111050791, LOC111051039, LOC111062967, LOC111063121, and LOC111054112. All the selected genes showed identical expression patterns as shown by DEGs analysis (S5 Fig), suggesting the reliability of RNA-seq analysis.
